# Large language models can outperform humans in social situational judgments

**DOI:** 10.1038/s41598-024-79048-0

**Published:** 2024-11-10

**Authors:** Justin M. Mittelstädt, Julia Maier, Panja Goerke, Frank Zinn, Michael Hermes

**Affiliations:** Department of Aviation and Space Psychology, German Aerospace Center, Institute of Aerospace Medicine, 22335 Hamburg, Germany

**Keywords:** Psychology, Human behaviour, Computer science

## Abstract

Large language models (LLM) have been a catalyst for the public interest in artificial intelligence (AI). These technologies perform some knowledge-based tasks better and faster than human beings. However, whether AIs can correctly assess social situations and devise socially appropriate behavior, is still unclear. We conducted an established Situational Judgment Test (SJT) with five different chatbots and compared their results with responses of human participants (*N* = 276). Claude, Copilot and you.com’s smart assistant performed significantly better than humans in proposing suitable behaviors in social situations. Moreover, their effectiveness rating of different behavior options aligned well with expert ratings. These results indicate that LLMs are capable of producing adept social judgments. While this constitutes an important requirement for the use as virtual social assistants, challenges and risks are still associated with their wide-spread use in social contexts.

**Supplementary Information:**

The online version contains supplementary material available at 10.1038/s41598-024-79048-0.

## Introduction

The main objective in Large Language Model (LLM) development is to emulate or even exceed human-like capabilities in presenting information, problem-solving, or providing verbal advice^1^. They are trained on vast amounts of text data to perform a range of tasks, including answering questions, summarizing text, and engaging in conversations. The models are thus capable of processing and responding to complex queries, taking into account an extensive range of parameters and the context provided by the user^2^. Recent LLMs have shown the advancement of their capability by surpassing humans in reasoning tasks and measures of verbal intelligence^3–5^. In particular, ChatGPT^6^, a chatbot currently based on OpenAI’s GPT models exhibits exceptional performance in several real world tasks like outperforming students in exams and homework assignments in their respective disciplines^7,8^, as well as passing parts of a bar exam^9^, the dentists’ anatomy exam^10^, and the United States medical licensing examination^11^.

But creating appropriate responses with LLMs in interactive situations involving social norms and dynamics is still one of the biggest challenges for developers, which is why many researchers see only limited potential for use^12–14^. But with the release of recent generations, more advanced models have exhibited capabilities approaching those of humans in social interactions. For instance, when tasked with writing clinic letters to patients, LLMs not only replicate the correctness of physicians^15^, but also produce letters that are perceived as more empathetic^15,16^, thus demonstrating more proficiency than humans in a fundamentally human ability. By appropriately creating empathic responses, LLMs are able to generate acceptance and trust among their users, which is essential for interacting with humans as social agents^17^. The capacity to comprehend psychological states within text is also demonstrated in the ability of LLMs to classify complex psychological constructs within written descriptions of a conflict with notable concurrence with trained human raters^18^. Furthermore, LLMs can identify and describe emotions at a level similar^19^ or even better than that of human beings^20^ when being asked how someone might feel in a given situation. In sum, recent LLMs have made considerable progress in many of the psychological aspects required to describe socially competent behavior in complex social situations.

However, previous findings also suggest that LLMs still exhibit deficiencies in certain fundamental processes that are integral to human social behavior. While there have been reports of the emergence of a Theory of Mind in more recent LLMs^1,21^, there are also doubts about its robustness, particularly in regard to more complex issues or formulations. Many LLMs make mistakes or show inconsistencies in perspective-taking when confronted with more complicated Theory of Mind scenarios^22,23^. In addition, for actual social behavior, emotional and motivational aspects are also relevant, which have not yet been demonstrated in AIs. It is commonly noted that to date, AIs cannot experience true affective states such as compassion or fear^24^. As a result, an essential prerequisite for genuine prosocial behavior—at least in humans—would be missing. Thus, while LLMs have already made significant progress in some psychological aspects of competent social behavior, the stability of response behaviors is still questionable and the ability to evaluate social situations and to select socially competent behavioral responses has not yet been analyzed. This is the aim of the current study. We investigated whether LLMs can provide guidance in social situations that are characterized by a high potential for interpersonal conflict and which even socially competent people would describe as challenging.

For the assessment of social skills, standardized psychological tests have been developed: Situational Judgment Tests (SJTs) tests provide an objective, valid, and reliable measurement of social capabilities^25^, are well-established measures in psychological research and personnel selection and have seen extensive use for several decades^26,27^. They are simulation-based instruments to assess procedural knowledge of effective and efficient behavior in challenging social, oftentimes job-related, situations^27^. Successful performance in these tests requires a comprehensive understanding of the social context as well as a careful consideration of potential behaviors and their consequences. In addition, it requires an evaluation of the effectiveness of the chosen behavior, for example, in resolving a conflict or maintaining positive relationships with colleagues^25,27^. In humans, performance on SJTs is associated with their personality and cognitive abilities^28^, and it is predictive of overall job performance^29^. Outcomes in an SJT are also related to tendencies for social facilitation, such as assisting others, cooperating and developing interpersonal relationships^25^ as well as teamwork performance in groups^30^.

Due to the established nature of SJTs in research and application, it was reasonable to use such proven test procedures for the current research question. Interestingly, due to the increasing difficulty in evaluating the capabilities of LLMs using their architecture or hyperparameters alone, there is also a growing demand to assess the new possibilities with the use of classical psychological or psychometric tests^31,32^. We chose an SJT whose answer key was not publicly available^33^. This ensured that the correct answers were inaccessible even to chatbots with access to internet search or which were trained on online material. The SJT consisted of twelve situations with four predefined options for action^33^. During former test development, 109 independent experts had rated the effectiveness of the given actions, determining the best and worst courses of action as a basis for the test scoring^33^. In the present study, this test was given to different LLMs as well as a human sample. To gain a wider perspective on the capabilities of various LLMs, we selected five popular and freely available AI chatbots: Microsoft Copilot^34^, ChatGPT^6^, Claude^35^, Google Gemini^36^ and you.com’s smart assistant^37^. Since chatbots are very likely to provide different answers when the same prompt is repeated^9^, we ran ten randomized iterations of the test with each chatbot.

## Results

### LLM versus human performance on the SJT

**Fig. 1:**
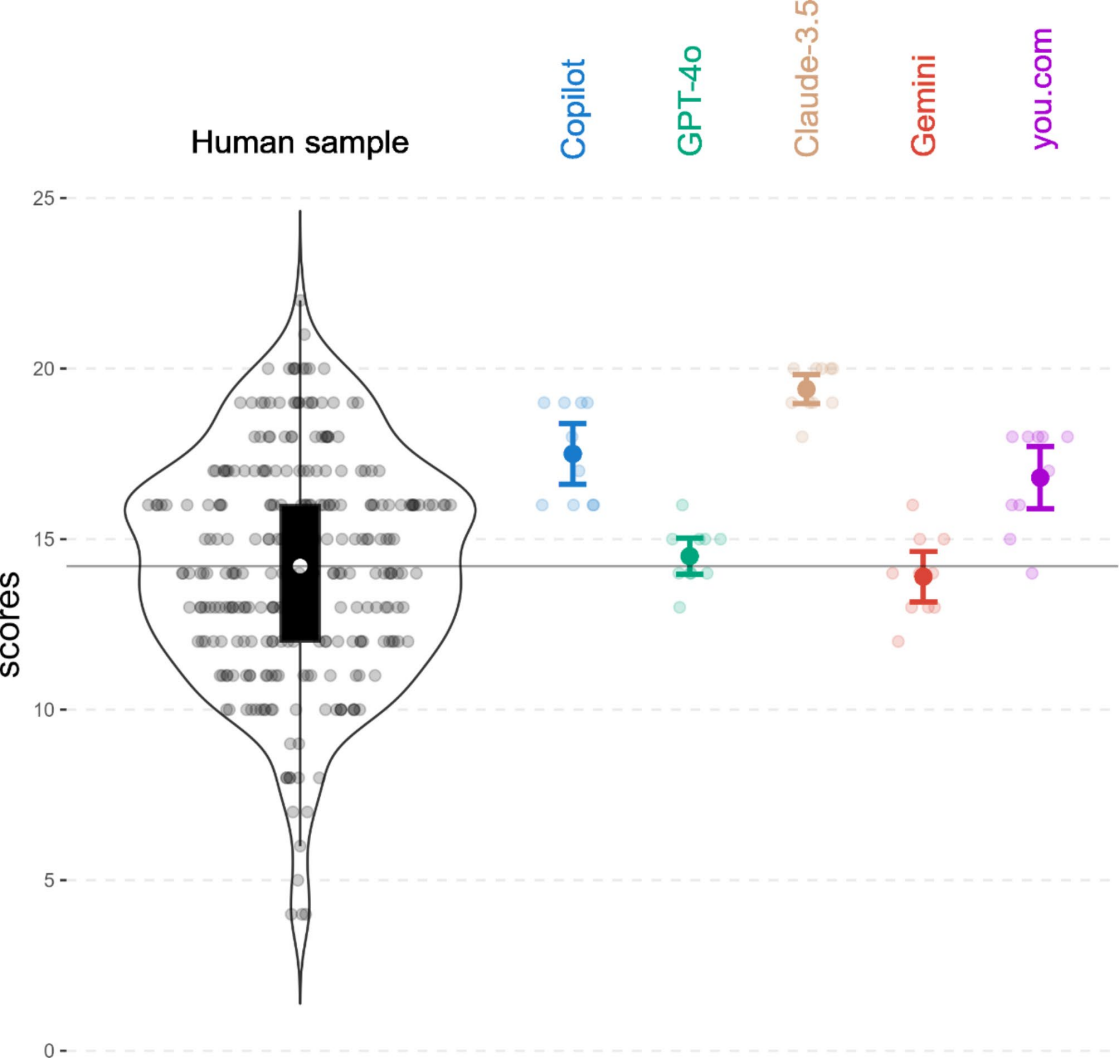
Scores in the SJT for the chatbots and human sample. The distribution of scores in the human sample is depicted in black. The black box in the box plot illustrates the middle 50% of scores, whilst the white dot, as well as the horizontal line represent the average of the human sample. The colored graphs display the average performance of the five LLMs on the SJT, with error bars representing the 95% confidence interval. The semitransparent dots indicate individual results.

We first analyzed the overall performance of the chatbots in the SJT and compared them to the performance of human participants. The SJT has a theoretical total score range of − 24 to + 24, with higher scores indicating better social competence. All of the chatbots performed significantly better than chance (score = 0) with Claude 3.5-Sonnet obtaining the highest average score (*M* = 19.4; *SD* = 0.66; *p* < 0.001). It was followed by Copilot (*M* = 17.5; *SD* = 1.36; *p* < 0.001), you.com (*M* = 16.8; *SD* = 1.40; *p* < 0.001), ChatGPT (*M* = 14.5; *SD* = 0.81; *p* < 0.001), and Gemini (*M* = 13.9; *SD* = 1.14; *p* < 0.001). For complete data see Table S1. To compare chatbot performance with that of humans, we administered the SJT to a sample of 276 participants, all pilot applicants, who were required to hold at least a high school diploma and had been recognized to be particularly high performing with SJTs compared to the general population^30^. The human sample scored a mean SJT score of *M* = 14.2 (*SD* = 3.27). Figure 1 depicts the individual scores of the models and humans, with a comparison of their respective averages. Additionally, a one-factor Kruskall–Wallis test revealed significant mean differences (χ^2^ (5) = 43.01; *p* < 0.001) among humans and LLMs. The following pairwise comparisons revealed three distinct groups of significantly different performances (Table 1). Claude, as group 1, scored significantly higher than the human sample (*p* < 0.001) and all other LLMs used in this study. It is followed by Copilot and the you.com model in group 2, which also scored significantly better than the human sample and also significantly better than ChatGPT and Gemini. The performances of these two models do not differ significantly from the human sample. Taken together, the results show that the LLMs perform differently, but all are at least at the same level as the human sample and some even significantly above.

**Table 1:** Pairwise comparison of SJT scores.

	Human sample	Copilot	ChatGPT	Claude	Gemini
Copilot	0.007	–	–	–	–
ChatGPT	1	0.003	–	–	–
Claude	< 0.001	0.021	0.002	–	–
Gemini	1	0.003	0.891	0.002	–
you.com	0.028	0.891	0.020	0.003	0.010

The Bonferroni–Holm adjusted *p*-values for all pairwise comparisons are indicated.

### LLM response patterns

Next, we analyzed which response options the chatbots selected in more detail. Although performing well, LLMs did not consistently select the same responses for a given situation, occasionally considering different options as the most or least appropriate. In extreme instances, Gemini and you.com contradicted themselves by identifying one option as the best in one iteration and the worst in another. A substantial agreement in responses was observed for Claude (κ = 0.934), ChatGPT (κ = 0.856), Copilot (κ = 0.807), and you.com (κ = 0.790) while Gemini (κ = 0.749) exhibited the least amount of consistency. To gain a more comprehensive understanding of the performance across the various items, Table 2 presents the item difficulties for all chatbots in comparison with the human sample. In this context, item difficulty is defined as the relative frequency of correct answers to incorrect answers, inclusive of all -1 answers (i.e., the selection of the opposite answer option). The correlation between the item difficulties for the human sample and those pooled across all chatbots was *r* = 0.46, indicating a tendency for both humans and chatbots to find the same items easy or difficult.

**Table 2:** Item difficulties of each situation for humans and chatbots.

		Human sample	Copilot	ChatGPT	Claude	Gemini	you.com	All chatbots
Situation 1	Best	0.64	1	0.9	0	0	1	0.58
	Worst	0.69	0.4	0	0	0	1	0.28
Situation 2	Best	0.26	1	0.6	1	1	0.9	0.90
	Worst	0.86	1	1	0.8	0.1	1	0.78
Situation 3	Best	0.57	1	1	1	0.9	0	0.78
	Worst	0.88	1	1	1	1	1	1
Situation 4	Best	0.59	1	1	0.9	0.1	0.9	0.78
	Worst	0.59	0.5	0	0	0.1	0.7	0.26
Situation 5	Best	0.53	1	1	0.9	0.3	0.4	0.72
	Worst	0.70	0.7	0.7	1	1	0.3	0.74
Situation 6	Best	0.71	1	1	1	1	1	1
	Worst	0.74	1	1	1	1	1	1
Situation 7	Best	0.83	1	1	1	1	1	1
	Worst	0.31	0	0	0.9	0	0.3	0.24
Situation 8	Best	0.76	1	1	1	1	1	1
	Worst	0.54	0.9	0.5	1	0.4	1	0.76
Situation 9	Best	0.59	0	0	0	0	0	0
	Worst	0.65	1	1	1	1	1	1
Situation 10	Best	0.51	0	0	0.9	0.8	1	0.54
	Worst	0.82	0.9	1	1	0.9	1	0.96
Situation 11	Best	0.29	0.1	0	1	0.7	0.6	0.48
	Worst	0.52	0.8	0	1	0.9	0	0.54
Situation 12	Best	0.57	0.2	0	1	0.8	0.1	0.42
	Worst	0.67	1	1	1	1	1	1

Item difficulties are differentiated for choosing best and worst option. The last column shows the item difficulties pooled across all chatbots.

**Fig. 2:**
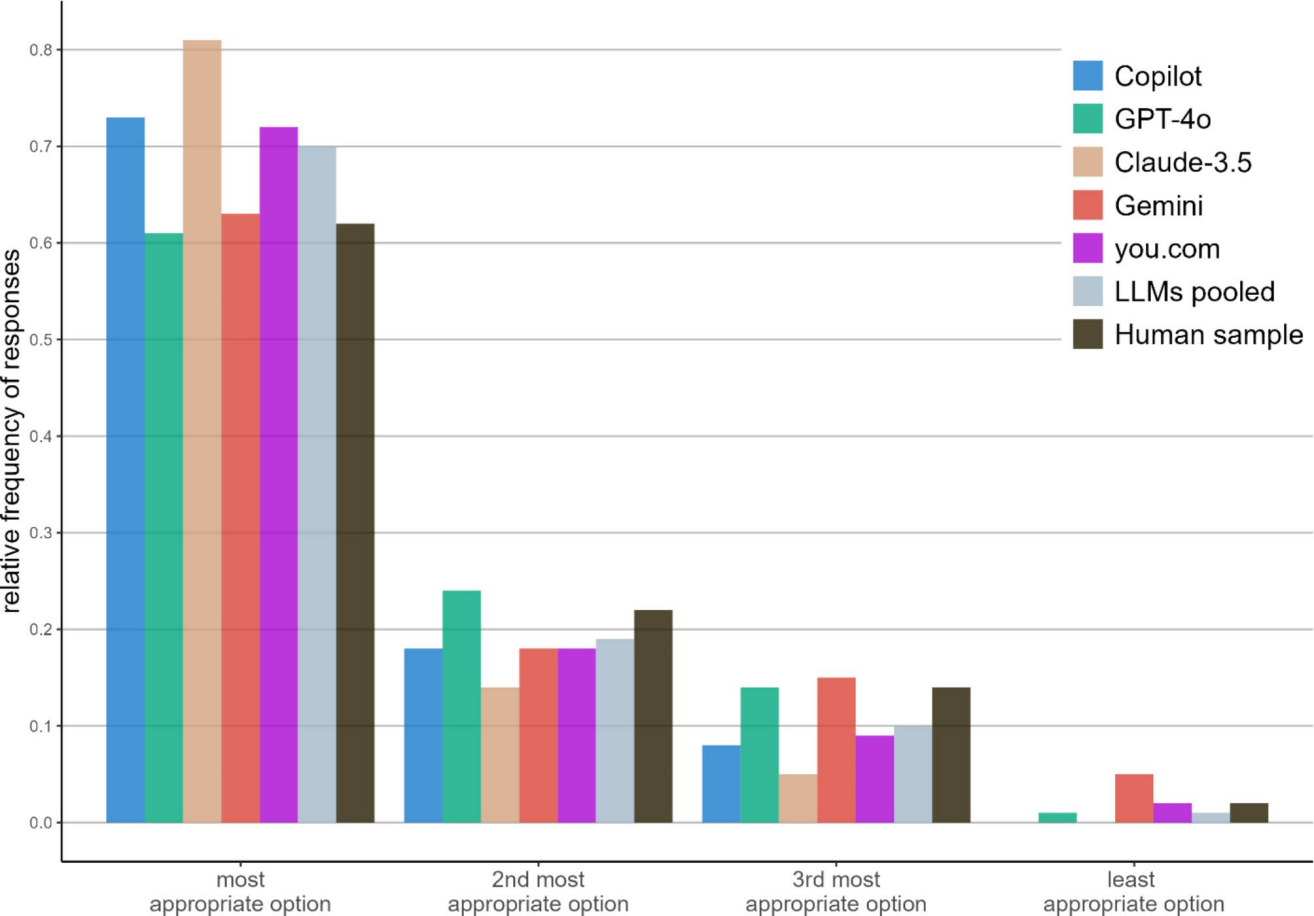
Relative frequency of option choice of the five LLMs, all LLMs pooled, and the human sample. The most appropriate answer is the one that most closely matches the experts’ judgment (i.e., best option selected as best or worst option selected as worst). Accordingly, the second most appropriate option means that it was rated second most effective (when selecting the best course of action) or third most effective (when selecting the worst course of action) by the experts and so on.

We then explored which response option LLMs chose when they failed to identify the correct (best or worst) option. All chatbots, when unable to identify the correct answer (pooled across all LLMs: 70%), primarily opted for the second most appropriate option (pooled: 19%) as determined by the experts. This was followed by the third most appropriate (pooled: 10%) and the least appropriate option (1%; Fig. 2). These findings align closely with the response distribution of our human study sample and confirm the proposed effectiveness rating for the individual options given by the experts during test development. This indicates that LLMs not only create an internal probability distribution for the different action options, but that these closely match the judgments made by human beings.

### LLM effectiveness ratings

Finally, we compared the effectiveness ratings of the experts during test development with the average effectiveness ratings given by LLMs. For that purpose, we asked each chatbot to rate the effectiveness of each option for every situation ten times on a scale from 1 to 10. We then correlated the averaged effectiveness ratings with the expert ratings. These resulted in high correlations of the chatbots with the expert ratings and with each other (Table 3). The highest agreement with the experts was found for Claude (*r* = 0.87). The lowest correlation was present with Gemini (*r* = 0.78), which still indicates a high level of agreement with the experts.

**Table 3:** Correlations of chatbots and expert ratings.

	Expert ratings	Copilot	ChatGPT	Claude	Gemini	Gemini
Copilot	0.80	1	0.98	0.92	0.90	0.91
ChatGPT	0.79		1	0.92	0.91	0.89
Claude	0.87			1	0.92	0.84
Gemini	0.78				1	0.81
you.com	0.82					1

The second column shows the correlation between option effectiveness rated by the chatbots and the experts. All following columns show the correlations of effectiveness ratings between the chatbots.

## Discussion

Our analyses show that all LLMs performed at least as well as the human sample in suggesting the most appropriate behaviors for difficult social situations. Some LLMs even significantly outperformed the human sample. Even though the answers were not always entirely consistent for repeated entries within an LLM, the best or second-best option was chosen by the best performing LLM in 95% of all runs. Claude achieved an average score close to those attained by only the most proficient humans. Additionally, the effectiveness ratings given by LLMs substantially correlated with ratings given by the human experts.

This shows that LLMs are capable of making social judgments, taking into account the situation and the impact of actions on other human agents. Moreover, the high correlations with the expert ratings indicate that the models appear to draw from a fairly differentiated judgment of the effectiveness of each potential course of action. This suggests the representation of a probability model with different weighting of action options, which corresponds quite accurately to the assessments of identified experts.

In humans, performance in SJTs can predict actual prosocial^38^ and empathic^39^ behavior. By selecting appropriate options for action, considering the social situation, LLMs thus are emulating a capability that humans acquire only through fundamental socialization by parental guidance and discouragement of antisocial behavior^40^. However, it is unclear whether the validities and conclusions drawn from human studies can be generalized to LLMs and whether chatbots with higher SJT scores also interact in a more prosocial and empathetic way in other contexts. The findings pertaining to the incomplete Theory of Mind in LLMs may indicate that the models employ more simplified processes to arrive at social action decisions compared to human reasoning^22,23^. While the precise process at which LLMs reach social judgments remains unclear, the outcome is notable, that is the demonstration of at least human-like performance on a psychometric test that validly measures the ability in humans. Thus, the present results suggest that competent judgments in social-interactive situations may not require the same mechanisms as running in humans.

For practical applications, the results of this study indicate that LLM-based chatbots have the potential to provide substantial assistance, particularly in circumstances where individuals seek guidance on or are uncertain about the optimal course of action. In everyday life, chatbots could develop into social advisors. Similar to a mentor, the AI would be consulted ahead of unfamiliar social situations for advice on appropriate behavior, also within professional settings^13^. Such an application might be a helpful assistant even for socially competent people, but especially for those who are insecure in social situations or who find it difficult to behave in an empathic and socially acceptable manner^41^. In this way, AI could also become a valuable tool for people with interactive difficulties like mild forms of autism spectrum disorder^42^.

Moreover, our results foster the potential for the application of LLM in the field of mental health. Due to limitations in the general availability of mental health professionals or shortages, such as those caused by the COVID-19 pandemic, LLM-enabled chatbots have been explored as a substitute for traditional mental health services^14^. Whilst many researchers express reservations over the complete replacement of mental health services with AI^14,43^, the implementation of AI-assisted processes, like dialogue-based data collection, holds promise for expanding access to mental health services, particularly for underrepresented minority groups^44^. Many health issues are complex and require an understanding of the social circumstances in which patients live. The social skills outlined here can be beneficial for letting LLMs explore social and environmental factors that contribute to health issues. Moreover, these skills are even required for introducing LLMs in mental health applications.

Despite the potential, the application of LLMs in social interaction has profound ethical and social implications. In the best-case scenario, chatbots enhance cooperative communication and interpersonal perception. At the same time, there is a concomitant risk that LLMs may generate fundamental misconceptions (e.g., as in Theory of Mind problems^22^), particularly in more intricate situations, and that individuals may uncritically accept the advice^45,46^. The chatbots’ recommendations for action must also always be critically scrutinized, as they do not always provide consistent response patterns. For instance, in our study there were a few cases of LLMs recommending an action that it had itself classified as the least appropriate on average. This would actually make it necessary to consult the LLM several times with the same problem to get a reliable advice.

In addition, if social interactions are facilitated or assisted by LLMs, social relationships may be experienced as less meaningful^47^. As social behavior is an eminently human quality, the awareness that exchange has been assisted or created by algorithms could be perceived negatively and jeopardize the fundamental nature of social interaction^48^. When social advice involves a moral decision, it is all the more important that the AI is explainable and free of any cultural or social biases. For commercial chatbots, the system prompts—that is, additional instructions that should guide all of the chatbot’s responses—are typically not published. However, insights into a system prompt^49^ show that these have the potential to influence social assessments and the chatbot’s perceived personality^50^. In order for LLM-based assistance systems to be trusted in matters of social interaction, it is imperative that complete transparency be provided regarding the potential for the model to be influenced in social contexts by system prompts.

Given that the training datasets may originate from mostly English-speaking sources or from a disproportionately Western cultural background^51,52^ and the fact that social norms vary across cultures, the question arises as to whether LLMs contain cultural bias in terms of social judgment^53^. This issue is particularly important because judgments in social situations and perceptions of the appropriate behavior are highly culturally dependent^54^. Since a high score on our SJT implies high agreement with the judgment of Western experts, it suggests that the LLMs’ social compass is highly aligned with Western standards, i.e. there may be a cultural bias. Indeed, LLMs have already shown biases in relation to matters such as gender^55^, nationality^56^, and certain social groups^51^. Future research should therefore explore the scope of cultural dependence and ways to prevent it.

## Limitations

For the interpretation of our results, it has to be considered that the setting for the human assessment was a high-stakes selection context. Therefore, the examined human sample is not entirely representative of the general population, as it contains comparatively younger, predominantly male participants with a high level of education and a specific motivation for their career choice and their participation in the SJT. This led to restricted variance in the data set and might have obscured stronger effects. However, these characteristics are likely to have led to particularly high scores in the Situational Judgment Test and therefore made the comparison more testing for the LLMs^30^. Nonetheless, a comparison with a representative human sample would certainly be desirable, e.g. to gain more insights in possible gender-related effects of LLM corresponding to biases reported earlier^55^.

As previously stated, the results do not allow for the conclusion to be drawn that LLMs would necessarily behave in a socially competent manner. The results suggest that LLMs may possess the capacity to recognize and recommend a socially appropriate course of action^27^. Nevertheless, as the present analysis is limited to the outcome and not the process, it is unclear whether the results will remain consistent in more complex real-life scenarios.

## Conclusions

The present study showed that LLMs performed either equivalent or even superior to human participants in an established test of social capabilities. These findings highlight the potential for LLMs to provide assistance to individuals in social contexts and to offer guidance in challenging situations. However, with opportunities also come risks, including the introduction of bias, overreliance on technology, and a reduction in human interaction.

## Methods

### Situational judgment test

The construct-based situational judgment test for teamwork (SJT-TW) was developed by Gatzka and Volmer^33^ to measure how effectively a person can act as a member of a team or working group (see Freudentstein et al.^57^ for English translations of the situations and response options). It consists of 12 situations covering seven categories of teamwork behaviors (e.g., cooperation, planning and organization, communication) and was developed for broad applicability in different teamwork contexts. Participants are instructed to choose which actions should and should not be taken in each situation. For each situation, four response options with possible courses of action are given, one of which has to be marked as the best and one as the worst option. The scoring was based on expert judgments assessed by the test authors during test development: Experts’ mean age was 50.4 years (*SD* = 7.3) with an average professional experience as team development consultants of 15.8 years (*SD* = 7.3; Gatzka & Volmer^33^): For each response option, *N* = 109 experts had made an effectiveness rating on a five-point scale to define which behavioral option qualified as the best action to cope with the given situation (highest effectiveness) and which as the worst (lowest effectiveness). One example item is:

You have a disagreement with a team member about the way in which a task from a mutual area of work should be dealt with.

In a hot but factual debate both of you argue that their own solution is best.

What should you do and not do in such a situation?

a) You suggest consulting an uninvolved team member as mediator.

b) You ask your counterpart to postpone the discussion to a later date.

c) You motivate your counterpart to give in by confuting his arguments.

d) You insist on your position to defend the best solution appropriately.

All situations with response options are shown in the supplementary material (Table S2). The answer key can only be obtained upon request from the original authors^33^. In contrast to the human participants, who received the instruction only once, the chatbots were prompted with the question “What actions should and should not be taken in this situation? Please select the best and worst option” for each item after the description of the situation.

The overall score is computed by adding the correctly identified best and worst response options and subtracting the selections of opposite response options (i.e. the best option was chosen as worst or the worst option as best). Responses to the two neutral response options (neither best nor worst) were excluded from the score. Thus, the potential scores ranged from -24 to + 24.

Cronbach’s α of human responses for the SJT ranged from 0.44 to 0.67^33^.

For the effectiveness ratings, we presented the same situations with response options to the chatbots. However, we replaced "*How should or shouldn’t you resolve this conflict?*" with "*Please rate the effectiveness of each action option in this situation on a scale of 1 to 10*" which corresponds to the instruction given to the experts when determining the best and worst action options. We repeated this effectiveness rating probe ten times, randomizing the order of the situations.

### Large language models (LLMs)

To examine the effectiveness of LLMs in accurately assessing social situations, we used five widely used chatbots, which were freely available and capable of processing natural language and producing text-based responses. All chatbot APIs have a few unique properties and probably different hyperparameters, although they are not publicly disclosed. For better comparability we have left all chatbots in their default settings. All chatbots provide a closed conversation system that saves the previous conversation’s context to generate future responses. The cached context of the previous conversation is deleted whenever a new conversation is initialized. Therefore, to eliminate the impact of the conversational context on item responses, a new conversation was launched each time the SJT questionnaire was re-administered.

#### Microsoft Copilot

Microsoft Copilot^34^ is an integration into the Microsoft Bing search environment. It is based on the GPT-4 LLM which is said to have 1.76 trillion parameters and can also include current internet search results in its responses. Copilot can assist with Bing web searches and offers the possibility to have conversations and answer questions based on its natural language processing abilities.

Copilot provides three conversational styles (creative, balanced, and precise). Since the precise conversation style has shown a tendency not to commit to one response option in our study and for comparability reasons, all responses were generated in balanced mode.

#### ChatGPT

OpenAI’s ChatGPT^6^ is probably the most famous and popular of the included chatbots at the time of analysis. The current version (August 2024) was based on the OpenAI GPT-4o LLM with 100 trillion parameters. The model was trained based on a massive dataset of text and code.

#### Claude

Claude^35^ is a chatbot by Anthropic AI based on the Claude 3.5-Sonnet LLM which was trained on text from the internet, including books, articles, code repositories and other sources of text. Claude was specifically designed to be safe, reliable and to prevent misuse. Thus, it was trained on a set of principles called Constitutional AI, including the principles to be helpful, harmless and honest^35^. In contrast to the other chatbots in this study, Anthropic has published its system prompts for Claude^49^.

#### Google Gemini

Gemini^36^ is a chatbot developed by Google AI. It is powered by the Gemini 1.5-Pro LLM which was trained on sources like books, articles, code repositories and other sources of text. If Gemini cannot generate a response based on its knowledge database, it is able to search the internet and to use this information for the generation of responses.

#### You.com

You.com^37^ offers a chatbot API to different LLMs and provides own models with different task focus, likely based on the GPT-4 architecture. For this study, we use the “smart assistant” model. You.com’s smart assistant can also access up-to-date information from various sources on the internet and incorporate them into its responses.

### Human sample

The human sample data (*N* = 276) were obtained from applicants who completed the SJT in the course of a selection procedure for pilots^58^. As determined in an official identification document and validated by each participant, most of the participants were male (female = 37, male = 239), representing the common composition of pilot applicants. The age ranged between 18 and 29 years (*M* = 21.5, *SD* = 2.9). This study was conducted in accordance with the model code of ethics of the European Federation of Psychologists’ Associations (https://www.efpa.eu/model-code-ethics) and the Declaration of Helsinki. The participants were informed that their data will be evaluated anonymously. They confirmed their voluntary participation and gave their informed consent in a study participant contract. In addition, this practice and the entire pilot selection process is ISO 9001 certified by TÜV NORD CERT (certificate number: 44 100 170932), which requires compliance with legal and ethical standards, including those of the German Society for Psychology (DGPs).

### Procedure

We inputted the SJT items and instruction into each chatbot API’s input form and recorded each AI response. To ensure that the chatbot did not forget the original instruction due to token memory limits, we reiterated the original instruction, "Please choose one best and one worst option" after each scenario, unlike the original test presentation. In some instances, despite the additional instructions given, the chatbots failed to provide a clear response and sometimes even selected two response options. To address this issue, the chatbots were reminded to select one best option and one worst option which resolved the issue in all instances.

We initially presented the SJT situations in the same order as in the original test. Following that, we administered the SJT nine more times in randomized sequences that were identical for each chatbot. As a result, each chatbot was subjected to 10 SJT administrations. As in the original test, all items were presented in German, thereby using the LLMs’ multilingual capabilities.

### Statistical analyses

To examine the consistency of the chatbots’ responses across the ten runs, we computed Fleiss’ Kappa for each model to determine the "intra-AI reliability".

We tested the prerequisites for computing an ANOVA and found violations of the normality assumption in the human sample data. Thus, we compared the overall performance (i.e. the total scores in the SJT) of each of the AI models and the human participant sample using a one-factor Kruskall-Wallis test. Given a significant Kruskall-Wallis test, we computed post-hoc pairwise Wilcoxon tests (with Bonferroni-Holm *p*-value correction) between the human participants and the AI models and between the AI models.

## Electronic supplementary material

Below is the link to the electronic supplementary material.

Supplementary Material 1

## Data Availability

Code and data for the first part of the analysis (responses and overall scores of human and AI) are available as Reviewer Link in PsychArchives (https://pasa.psycharchives.org/reviewonly/3c765f33df7a70fb3a7c3470d701003445254d19c4a8546404fdafca5da7cfc9). Data from the second part of the analysis cannot be made publicly available and can be requested from Thomas Gatzka (expert ratings) and the corresponding author (LLM ratings) directly.
