# Supplementary material for: Large language models can outperform humans in social situational judgments
Source: Sci Rep. 2024 Nov 10;14:27449. doi: 10.1038/s41598-024-79048-0 (PMC11551142; doi:10.1038/s41598-024-79048-0)
Supplement: Supplementary file 1 — Supplementary Material 1 [file 41598_2024_79048_MOESM1_ESM.docx]

**Supplementary material**

**Table S1. Overview of the selected response options of the LLMs.** B means the best response option, while W is the worst response option. N1 and N2 are the two neutral response options. Correctly identified options are in bold and wrong answers in italic.

|  |  | Copilot | | | | | | | | | | ChatGPT | | | | | | | | | | Claude | | | | | | | | | | Gemini | | | | | | | | | | | you.com | | | | | | | | | | |
| --- | --- | --- | --- | --- | --- | --- | --- | --- | --- | --- | --- | --- | --- | --- | --- | --- | --- | --- | --- | --- | --- | --- | --- | --- | --- | --- | --- | --- | --- | --- | --- | --- | --- | --- | --- | --- | --- | --- | --- | --- | --- | --- | --- | --- | --- | --- | --- | --- | --- | --- | --- | --- | --- |
|  |  | 1 | 2 | 3 | 4 | 5 | 6 | 7 | 8 | 9 | 10 | 1 | 2 | 3 | 4 | 5 | 6 | 7 | 8 | 9 | 10 | 1 | 2 | 3 | 4 | 5 | 6 | 7 | 8 | 9 | 10 | 1 | 2 | 3 | 4 | 5 | 6 | 7 | 8 | 9 | 10 | 1 | | 2 | 3 | 4 | 5 | 6 | 7 | 8 | 9 | 10 |  |
| Sit. 1 | best | **B** | **B** | **B** | **B** | **B** | **B** | **B** | **B** | **B** | **B** | **B** | **B** | **B** | **B** | *W* | **B** | **B** | **B** | **B** | **B** | N1 | N1 | N1 | N1 | N1 | N1 | N1 | N1 | N1 | N1 | *W* | *W* | *W* | N1 | N1 | N1 | *W* | *W* | *W* | *W* | **B** | | **B** | **B** | **B** | **B** | **B** | **B** | **B** | **B** | **B** |  |
|  | worst | N2 | N2 | N2 | N2 | N2 | **W** | **W** | **W** | N2 | **W** | N2 | N2 | N2 | N2 | N2 | N2 | N2 | N2 | N2 | N2 | N2 | N2 | N2 | N2 | N2 | N2 | N2 | N2 | N2 | N2 | N2 | N2 | N2 | N2 | N2 | N2 | N2 | N2 | N2 | N2 | **W** | | **W** | **W** | **W** | **W** | **W** | **W** | **W** | **W** | **W** |  |
| Sit. 2 | best | **B** | **B** | **B** | **B** | **B** | **B** | **B** | **B** | **B** | **B** | N2 | **B** | N2 | N2 | **B** | N2 | **B** | **B** | **B** | **B** | **B** | **B** | **B** | **B** | **B** | **B** | **B** | **B** | **B** | **B** | **B** | **B** | **B** | **B** | **B** | **B** | **B** | **B** | **B** | **B** | N2 | | **B** | **B** | **B** | **B** | **B** | **B** | **B** | **B** | **B** | |
|  | worst | **W** | **W** | **W** | **W** | **W** | **W** | **W** | **W** | **W** | **W** | **W** | **W** | **W** | **W** | **W** | **W** | **W** | **W** | **W** | **W** | **W** | **W** | N1 | **W** | **W** | **W** | **W** | **W** | N1 | **W** | N1 | N1 | N1 | N1 | **W** | N1 | N1 | N1 | N1 | N1 | **W** | | **W** | **W** | **W** | **W** | **W** | **W** | **W** | **W** | **W** | |
| Sit. 3 | best | **B** | **B** | **B** | **B** | **B** | **B** | **B** | **B** | **B** | **B** | **B** | **B** | **B** | **B** | **B** | **B** | **B** | **B** | **B** | **B** | **B** | **B** | **B** | **B** | **B** | **B** | **B** | **B** | **B** | **B** | **B** | **B** | **B** | **B** | **B** | N1 | **B** | **B** | **B** | **B** | N1 | | N1 | N1 | N1 | N1 | N1 | N1 | N1 | N1 | N1 | |
|  | worst | **W** | **W** | **W** | **W** | **W** | **W** | **W** | **W** | **W** | **W** | **W** | **W** | **W** | **W** | **W** | **W** | **W** | **W** | **W** | **W** | **W** | **W** | **W** | **W** | **W** | **W** | **W** | **W** | **W** | **W** | **W** | **W** | **W** | **W** | **W** | **W** | **W** | **W** | **W** | **W** | **W** | | **W** | **W** | **W** | **W** | **W** | **W** | **W** | **W** | **W** | |
| Sit. 4 | best | **B** | **B** | **B** | **B** | **B** | **B** | **B** | **B** | **B** | **B** | **B** | **B** | **B** | **B** | **B** | **B** | **B** | **B** | **B** | **B** | **B** | **B** | **B** | **B** | **B** | **B** | **B** | **B** | N1 | **B** | N1 | N1 | *W* | N1 | **B** | N1 | *W* | N1 | N1 | *W* | **B** | | **B** | **B** | **B** | N2 | **B** | **B** | **B** | **B** | **B** | |
|  | worst | N2 | **W** | N2 | **N2** | N2 | **W** | **W** | **W** | N2 | **W** | N2 | N2 | N2 | N2 | N2 | N2 | N2 | N2 | N2 | N2 | N2 | N2 | N2 | N2 | N2 | N2 | N2 | N2 | N2 | N2 | N2 | N2 | N2 | N2 | **W** | N2 | N2 | N2 | *B* | N2 | **W** | | **W** | **N2** | **W** | **W** | N2 | **W** | N2 | **W** | **W** | |
| Sit. 5 | best | **B** | **B** | **B** | **B** | **B** | **B** | **B** | **B** | **B** | **B** | **B** | **B** | **B** | **B** | **B** | **B** | **B** | **B** | **B** | **B** | **B** | **B** | **B** | **B** | **B** | **B** | **B** | **B** | **B** | N2 | **B** | N2 | N2 | N2 | N2 | N2 | **B** | N2 | N2 | **B** | **B** | | N1 | *W* | N2 | **B** | *W* | *W* | **B** | **B** | N2 | |
|  | worst | **W** | **W** | **W** | N1 | **W** | **W** | **N1** | N1 | **W** | **W** | N1 | N2 | **W** | **W** | **W** | **W** | **W** | **W** | N1 | **W** | **W** | **W** | **W** | **W** | **W** | **W** | **W** | **W** | **W** | **W** | **W** | **W** | **W** | **W** | **W** | **W** | **W** | **W** | **W** | **W** | N1 | | N2 | N1 | N1 | **W** | *B* | N1 | **W** | N1 | **W** | |
| Sit. 6 | best | **B** | **B** | **B** | **B** | **B** | **B** | **B** | **B** | **B** | **B** | **B** | **B** | **B** | **B** | **B** | **B** | **B** | **B** | **B** | **B** | **B** | **B** | **B** | **B** | **B** | **B** | **B** | **B** | **B** | **B** | **B** | **B** | **B** | **B** | **B** | **B** | **B** | **B** | **B** | **B** | **B** | | **B** | **B** | **B** | **B** | **B** | **B** | **B** | **B** | **B** | |
|  | worst | **W** | **W** | **W** | **W** | **W** | **W** | **W** | **W** | **W** | **W** | **W** | **W** | **W** | **W** | **W** | **W** | **W** | **W** | **W** | **W** | **W** | **W** | **W** | **W** | **W** | **W** | **W** | **W** | **W** | **W** | **W** | **W** | **W** | **W** | **W** | **W** | **W** | **W** | **W** | **W** | **W** | | **W** | **W** | **W** | **W** | **W** | **W** | **W** | **W** | **W** | |
| Sit. 7 | best | **B** | **B** | **B** | **B** | **B** | **B** | **B** | **B** | **B** | **B** | **B** | **B** | **B** | **B** | **B** | **B** | **B** | **B** | **B** | **B** | **B** | **B** | **B** | **B** | **B** | **B** | **B** | **B** | **B** | **B** | **B** | **B** | **B** | **B** | **B** | **B** | **B** | **B** | **B** | **B** | **B** | | **B** | **B** | **B** | **B** | **B** | **B** | **B** | **B** | **B** | |
|  | worst | N1 | N2 | N2 | N1 | N1 | N2 | N1 | N1 | N1 | N2 | N1 | N1 | N1 | N1 | N2 | N2 | N1 | N1 | N1 | N2 | **W** | **W** | **W** | **W** | **W** | **W** | **W** | N1 | **W** | **W** | N1 | N1 | N1 | N1 | N1 | N1 | N1 | N1 | N1 | N1 | N1 | | N1 | N1 | N1 | N1 | **W** | N1 | **W** | N1 | **W** | |
| Sit. 8 | best | **B** | **B** | **B** | **B** | **B** | **B** | **B** | **B** | **B** | **B** | **B** | **B** | **B** | **B** | **B** | **B** | **B** | **B** | **B** | **B** | **B** | **B** | **B** | **B** | **B** | **B** | **B** | **B** | **B** | **B** | **B** | **B** | **B** | **B** | **B** | **B** | **B** | **B** | **B** | **B** | **B** | | **B** | **B** | **B** | **B** | **B** | **B** | **B** | **B** | **B** | |
|  | worst | **W** | **W** | **W** | **W** | N2 | **W** | **W** | **W** | **W** | **W** | **W** | **W** | N2 | N2 | **N2** | **W** | N2 | N2 | **W** | **W** | **W** | **W** | **W** | **W** | **W** | **W** | **W** | **W** | **W** | **W** | N2 | N2 | **W** | **W** | **W** | N2 | N2 | N2 | **W** | N2 | **W** | | **W** | **W** | **W** | **W** | **W** | **W** | **W** | **W** | **W** | |
| Sit. 9 | best | N1 | N1 | N1 | N1 | N1 | N1 | N1 | N1 | N1 | N1 | N1 | N1 | N1 | N1 | N1 | N1 | N1 | N1 | N1 | N1 | N1 | N1 | N1 | N1 | N1 | N1 | N1 | N1 | N1 | N1 | N1 | N1 | N1 | N1 | N1 | N1 | N1 | N1 | N1 | N1 | N1 | | N1 | N1 | N1 | N1 | N1 | N1 | N1 | N1 | N1 | |
|  | worst | **W** | **W** | **W** | **W** | **W** | **W** | **W** | **W** | **W** | **W** | **W** | **W** | **W** | **W** | **W** | **W** | **W** | **W** | **W** | **W** | **W** | **W** | **W** | **W** | **W** | **W** | **W** | **W** | **W** | **W** | **W** | **W** | **W** | **W** | **W** | **W** | **W** | **W** | **W** | **W** | **W** | | **W** | **W** | **W** | **W** | **W** | **W** | **W** | **W** | **W** | |
| Sit. 10 | best | N2 | N2 | N2 | N2 | N2 | N2 | N2 | N2 | N2 | N2 | N2 | N2 | N2 | N2 | N2 | N2 | N2 | N2 | N2 | N2 | **B** | **B** | **B** | **B** | **B** | **B** | **B** | **B** | **B** | **B** | **B** | **B** | **B** | **B** | N2 | **B** | **B** | **B** | **B** | N2 | **B** | | **B** | **B** | **B** | **B** | **B** | **B** | **B** | **B** | **B** | |
|  | worst | **W** | **W** | **W** | **W** | **W** | **W** | **W** | **W** | N1 | **W** | **W** | **W** | **W** | **W** | **W** | **W** | **W** | **W** | **W** | **W** | **W** | **W** | **W** | **W** | **W** | **W** | **W** | **W** | **W** | **W** | **W** | **W** | **W** | **W** | **W** | **W** | **W** | **W** | N1 | **W** | **W** | | **W** | **W** | **W** | **W** | **W** | **W** | **W** | **W** | **W** | |
| Sit. 11 | best | N1 | N1 | N1 | N1 | N1 | N1 | N1 | N1 | N1 | N1 | N1 | N1 | N1 | N1 | N1 | N1 | N1 | N1 | N1 | N1 | **B** | **B** | **B** | **B** | **B** | **B** | **B** | **B** | **B** | **B** | **B** | **B** | **B** | N2 | **B** | N2 | **B** | N2 | **B** | **B** | **B** | | N1 | **B** | **B** | **B** | N1 | **B** | N1 | **B** | N1 | |
|  | worst | N2 | **W** | **W** | **W** | **W** | **N2** | **W** | **W** | **W** | **W** | N2 | N2 | N2 | N2 | N2 | N2 | N2 | N2 | *B* | N2 | **W** | **W** | **W** | **W** | **W** | **W** | **W** | **W** | **W** | **W** | **W** | **W** | **W** | **W** | N1 | **W** | **W** | **W** | **W** | **W** | N2 | | N2 | N2 | N2 | N1 | N2 | N2 | N2 | N2 | N2 | |
| Sit. 12 | best | N2 | N2 | N2 | N2 | N2 | N2 | **B** | **B** | N2 | N2 | **N2** | **N2** | **N2** | **N2** | **N2** | **N2** | **N2** | **N2** | **N2** | **N2** | **B** | **B** | **B** | **B** | **B** | **B** | **B** | **B** | **B** | **B** | **B** | **B** | **B** | **B** | N2 | **B** | **B** | **B** | N2 | **B** | N2 | | N2 | N2 | **B** | N2 | N2 | N2 | N2 | N2 | N2 | |
|  | worst | **W** | **W** | **W** | **W** | **W** | **W** | **W** | **W** | **W** | **W** | **W** | **W** | **W** | **W** | **W** | **W** | **W** | **W** | **W** | **W** | **W** | **W** | **W** | **W** | **W** | **W** | **W** | **W** | **W** | **W** | **W** | **W** | **W** | **W** | **W** | **W** | **W** | **W** | **W** | **W** | **W** | | **W** | **W** | **W** | **W** | **W** | **W** | **W** | **W** | **W** | |
| Total score | | 16 | 19 | 17 | 16 | 16 | 18 | 19 | 19 | 16 | 19 | 14 | 15 | 14 | 14 | 13 | 15 | 15 | 15 | 14 | 16 | 19 | 20 | 19 | 20 | 20 | 20 | 20 | 19 | 18 | 19 | 15 | 14 | 14 | 15 | 16 | 13 | 14 | 13 | 12 | 13 | 17 | | 16 | 15 | 18 | 18 | 14 | 16 | 18 | 18 | 18 |  |

**Table S2. All twelve situations of the SJT with action options.** Listed are the translated items by Freudenstein et al.^57^, the human participants and the AI were presented the original items in German language by Gatzka & Volmer^33^.

| Item no | situation | response options |
| --- | --- | --- |
| 1 | You are temporarily subjected to personal stress that also affects your occupational activity. A briefly acquainted colleague asks you about the reason for your decline in performance and offers help with your task. | What should you do and not do in such a situation?   1. You confirm an increase in personal stress and accept the help. 2. You do not mention your personal problems, but accept the help. 3. You explain your specific situation and ask for help with your task. 4. You thank your colleague for the feedback but politely decline the offer. |
| 2 | Your team has clearly allocated all areas of responsibility. However, you incidentally notice that some team members in another area are challenged by a task that you have experience in. | What should you do and not do in such a situation?   1. You tell your colleagues about your experience and offer advisory support. 2. You mention your expertise and offer active support. 3. You ask whether your experience or advice is desired and if so, when. 4. Your respect your colleagues’ responsibilities and stay out of it. |
| 3 | Your team has made a lot of progress working on a complex task when some unforeseen developments occur. Therefore, your tediously achieved results are no longer completely up to date. | What should your team do and not do in such a situation?   1. Slight shortcomings will be tolerated due to the advanced progress of the work. 2. The team asks the customer or superior for their assessment of the situation. 3. Team members immediately discuss possible consequences in a meeting. 4. Changes are retrospectively implemented through intensive additional work. |
| 4 | You notice a sudden but continuous decrease in performance in one of your team members, whom you have experienced as competent and reliable. Other sources report that this colleague currently has some personal problems. | What should you do and not do in such a situation?   1. You and the other team members discuss how to support this colleague. 2. You respect your colleague’s privacy and do not get involved in private matters. 3. You help your colleague without asking questions. 4. You ask your colleague if they want to talk about their problems. |
| 5 | Some of your colleagues discuss various aspects of a team task during a meeting. Your area of responsibility is not the focus of their discussion, which is why you hold back and refrain from partaking in it. | What should you do and not do in such a situation?   1. You mentally prepare the discussion points that you wanted to address. 2. You use the opportunity to broaden your knowledge about other parts of the task. 3. You carefully steer the conversation towards a more familiar topic that you can engage in. 4. You attentively look for information that could be important for your area of responsibility. |
| 6 | You are transferred to an already existing team. During a brief introduction, your contact person tells you that all team members have their own area of responsibility. Without providing any further details, your contact person instructs you on your own area of responsibility. | What should you do and not do in such a situation?   1. You limit your questions to your area of responsibility, as nothing else should concern you. 2. You hold back your curiosity and carefully listen to your contact person’s explanations. 3. You decide to become familiar with the other areas of responsibility on your own after the conversation. 4. You ask for the basic workflows and interdependencies in the team. |
| 7 | You have to inform another team member about a complex issue from your area of responsibility. It is of utmost importance for your team’s success that the other person takes note of your concern and that no uncertainties are left. | What should you do and not do in such a situation?   1. You prepare a timesaving summary that you personally deliver. 2. You send a detailed report and ask for an acknowledgement of receipt. 3. You arrange a personal meeting with the other team member. 4. You send a message and explicitly request the other team member to contact you if any uncertainties are left. |
| 8 | You incidentally notice that another team member struggles to finish their work on time. You have already completed your tasks. However, you want to double check your work and, if necessary, improve some details before the deadline. | What should you do and not do in such a situation?   1. You ask the team member in a confidential conversation whether they need help. 2. You contain yourself because you do not want the team member to appear incompetent. 3. You carefully address your observation in the next team meeting. 4. You finish your own tasks first before offering your help. |
| 9 | Together with your team members, you are setting objectives for each member for an upcoming task. | What should your team do and not do in such a situation?   1. The team sets objectives that are positive, clearly defined and easily verifiable. 2. The team sets objectives that are specific, challenging and agreed upon by the whole team. 3. The team sets objectives that are moderately difficult and comprehensible to the whole team. 4. The team sets objectives that are easily attainable, open and flexible concerning time management. |
| 10 | You are working on a task that is mainly in your area of responsibility. When you present your intended procedure during a meeting, some team members from other areas speak up and add suggestions for changes and adaptations. | What should you do and not do in such a situation?   1. You take note of suggestions and discuss them with everyone involved. 2. You reflect on what changes might be sensible and ask for details. 3. You politely point out that you have a better overview of the task due to your expertise. 4. You try to include as many of the suggested changes as possible in your plan. |
| 11 | Due to external circumstances, your team was unable to finish an important task on time. Since every team member has given their very best, there is considerable disappointment. When a new task comes up, you notice that low morale and poor motivation are impairing the team. | What should you do and not do in such a situation?   1. You remind your team of past successes to spark new motivation. 2. You address your concerns in front of the whole team and encourage a discussion. 3. You ask for a team meeting to put the failure behind you. 4. You give the other team members the time to regain their motivation. |
| 12 | Together with your team members, you are planning how to tackle an upcoming task. The team’s success in mastering this challenge depends on several factors, some of which are difficult to predict. | What should your team do and not do in such a situation?   1. The team discusses all possible developments in advance and works out a strategy for each of them. 2. The planning proceeds in small steps in order to allow quick adaptations. 3. The team waits with further planning until all uncertainties are eliminated. 4. The team focuses especially on currently available facts for the planning. |
